# Patchy and Pink: Dynamics of a *Chlainomonas* sp. (*Chlamydomonadales*, chlorophyta) algal bloom on Bagley Lake, North Cascades, WA

**DOI:** 10.1093/femsec/fiad106

**Published:** 2023-09-07

**Authors:** Dan van Hees, Clare Hanneman, Sophie Paradis, A G Camara, Maya Matsumoto, Trinity Hamilton, Stacy A Krueger-Hadfield, Robin B Kodner

**Affiliations:** Biology Department, Western Washington University, Bellingham, WA 98225, United States; Biology Department, Western Washington University, Bellingham, WA 98225, United States; Biology Department, Western Washington University, Bellingham, WA 98225, United States; Biology Department, Western Washington University, Bellingham, WA 98225, United States; Biology Department, Western Washington University, Bellingham, WA 98225, United States; Department of Plant and Microbial Biology and the BioTechnology Institute, University of Minnesota St. Paul, MN 55108, United States; Department of Biology, The University of Alabama at Birmingham, Birmingham, AL 35294, United States; Environmental Science, Western Washington University, Bellingham, WA 98225, United States

**Keywords:** *Chlainomonas* sp, snow algae, blooms, dynamics, photophysiology

## Abstract

Snow algal blooms frequently occur throughout alpine and polar environments during spring and summer months; however, our understanding of bloom dynamics is limited. We tracked a recurrent bloom of *Chlainomonas* sp. on Upper Bagley Lake in the North Cascade Mountains, USA, to assess the spatiotemporal dynamics in bloom color intensity, community photophysiology, and community composition over eight weeks. We found that the algae biomass had a dynamic patchy distribution over space and time, which was decoupled from changes in community composition and life-cycle progress averaged across the bloom. The proportional representation of *Chlainomonas* sp. remained consistent throughout the study while the overall community composition shows a progression through the bloom. We found that community photophysiology, measured by the maximum quantum yield of PSII (Fv/Fm), decreased on average throughout the bloom. These findings suggest that the *Chlainomonas* sp. community on Bagley Lake is not simply an algal bloom with rapid increase in biomass followed by a population crash, as is often seen in aquatic systems, though there is a physiological trajectory and sensitivity to environmental stress. These results contribute to our understanding of the biology of *Chlainomonas* sp. and its response to environmental stress, specifically an extreme warming event.

## Teaser

A weekly charactrization of an 8-week bloom of the snow algae, *Chlainomonas* sp., in a snow-on-lake progressed in asynchronous patches that showed a stress response via photophysiology measurments to an extreme heat event.

## Introduction

Snow algae, a group of closely related species in the phylum Chlorophyta, are adapted to live and bloom in snow habitats and grow and photosynthesize at near-freezing temperatures. Algal biomass can turn snow surfaces pink, red, orange, and green during bloom events due to the dominant pigments in the cells. These blooms are important in the cryosphere, because they reduce the reflectance of solar radiation off snow surfaces, which accelerates snowmelt in snowpacks and glaciers that are currently already melting rapidly due to climate warming (Yallop et al. 2012, Ganey et al. 2017, Hotaling et al. 2021). Snow algal blooms are a seasonal phenomenon and only appear in spring and summer months when there is liquid water available in snow to facilitate growth and reproduction (Hoham 1974a, Hoham and Mullet 1977, Ling and Seppelt 1998, Hoham 2000, Kvíderová 2010). Though the seasonal appearance of snow algae in polar and alpine snow habitats reliably happens every year, there are still many open questions about the mechanisms by which snow algae repopulate the snow annually and how blooms initiate.

Though no single parameter is used to define what is considered a “snow algal bloom,” it is generally known to be an event that is defined by relatively high biomass concentrations compared to seasonal averages and is typically time-limited (Smayda 1997). In aquatic systems, blooms are understood to be events that produce abundant biomass that appears in a relatively short period of time. In contrast, the persistence of high biomass can have variable durations (Sverdrup 1953). In snow, algal blooms are typically described as the appearance of visible biomass on snow surfaces that turns the snow pink, red, orange, or green due to the abundant pigments found in cells. There is a connection between the intensity of color on snow surfaces and snow algae biomass, where higher biomass produces darker colors. However, this relationship is not well characterized or constrained. In places where snow algae bloom on seasonal snowpack, biomass often persists until the habitat disappears or until it is buried by new snow accumulation at the end of the growing season.

Despite the well-documented phenomenon of snow algae blooms on snow surfaces worldwide in alpine and polar environments, characterization of bloom progression across a growing season is limited. There are two primary ways to characterize the progression of snow algal blooms over time: (i) observation of color (i.e., biomass) on snow surfaces and (ii) characterization of the snow algal biology via microscopy, physiology, molecular tools, and other biological methodologies. Because snow algal blooms are so visible on snow surfaces, observation and quantification of color can be used as a measure of bloom area and can be correlated with snow algal biomass. Many kinds of remote sensing technologies have been used to observe snow algal blooms on snow surfaces, in an attempt to quantify bloom size and impact on melt (Painter et al. 2001, Gray et al. 2021, Engstrom et al. 2022). Recently, remote sensing has been used in the Pacific Northwest region of North America to show changes in snow algal biomass over time on glaciers (Engstrom et al. 2022). While remote sensing methods are just starting to attempt to link snow algal presence and ecological drivers of blooms (Gray et al. 2021), these methods work at large, landscape scales, and the fundamental unit of a bloom is microscopic cells. Though we still have very little understanding of what controls snow algae bloom onset and dynamics, it's very likely blooms may be controlled at much smaller scales (on the order of meters or centimeters), especially when blooms are patchy as is often seen in alpine snow that is not associated with large ice fields/glaciers. These types of alpine snow habitats can be particularly complex with many microhabitats such as tarns, lakes, talus, vegetation, soil, and gravel, underlying snow. Though large remote sensing studies are helpful in predicting the albedo consequences of snow algal blooms, they currently do little to help us understand the biological dynamics that ultimately support the onset of and control blooms, particularly in patchy and complex snow habitats.

The biological dynamics of snow algal blooms can be described in terms of biodiversity, physiology, and ecological dynamics throughout a bloom. While there are upward of 40 described taxa of algae adapted to live in snow (Hoham and Remais 2020), there are only a few species from three genera — *Sanguina* (formerly assigned to *Chlamydomonas*), *Chlainomonas*, and *Chloromonas* that produce visible pink or red blooms in snow. Thus, biodiversity in snow is relatively simple compared to most aquatic systems. Our understanding of snow algae biodiversity has been improving recently with the use of gene-based surveys (Lutz et al. 2016, Segawa et al. 2018, Engstrom et al. 2020). Biodiversity surveys in Pacific Northwest pink and red snow blooms suggest blooms can be dominated by the snow algae genera *Sanguina, Chlainomonas*, or *Chloromonas* and that the composition of blooms changes across elevation and season (Mallon 2019, Engstrom et al. 2020). The composition of other microbes and microeukaryotes that interact with the algae show can also vary by location and season (Lutz et al. 2016, Tucker and Brown, 2022).

Snow algae physiology has been studied across many environments and species using different measures of photophysiology. Photophysiology can be used to track changes in the algae's ability to do photosynthesis through time as environmental conditions shift through the duration of a bloom (Stibal et al. 2007, Tala et al. 2017). Pulse Amplitude Modulated (PAM) fluorometry is a rapid and non-invasive tool has been used extensively throughout macroalgal marine environments to study algal photophysiology (Tala et al. 2017, Gao et al. 2018) as well as in arctic (Kuhl et al. 2001) and alpine algal environments (Kvíderová 2010, Stibal et al. 2007, Procházková et al. 2018) to address natural community photophysiology. PAM fluorimetry can be particularly useful when used to measure the maximum quantum yield of PSII (Fv/Fm), which is widely used in study of algae as a metric that can be affected by stress, including environmental stress like temperature and light (Schreiber 2004, Zheng et al. 2020).

Snow algae, like all green algae, have complex life cycles and alternate between stages with different morphologies, and presumably different ploidies, using a combination of sexual and asexual (e.g., mitotic divisions) reproduction that may impact how and when they bloom (Hoham and Ling 2000). Often, algae that produce harmful blooms in marine systems will undergo rapid asexual growth to produce high biomass quickly, and then resting spores once resources are exhausted (Smayda 1997). *Chlainomonas* and *Sanguina*, two of the most common red snow producing taxa, cannot currently be cultured in the laboratory. Thus, making connections between life cycle transitions and bloom formation in these taxa is challenging. Snow algal life cycles have been well described in many snow chloromonads species, but these taxa are not known to produce large blooms across landscape scales and do typically not produce large red blooms (Hoham and Remias 2020). What is known about the life cycles of the red blooming taxa, *Sanguina* and *Chlainomonas*, comes primarily from field-collected samples. A recent study has reported a flagellate stage of *Sanguina*, but little is known about the life cycle of this abundant snow alga (Raymond et al. 2022). *Chlainomonas*, in contrast, has a dynamic life cycle with many morphological stages described from field collections from multiple species and found across the world, including North America, Europe, Japan, and New Zealand (Hoham 1974a, Hoham 1974b, Novis 2002b, Novis et al. 2008, Remias et al. 2016, Procházková et al. 2018, Matsuzaki et al. 2022, Matsumoto et al. in review).

In addition to characterizing the snow algae themselves during a bloom, it is critical to understand how snow habitat changes throughout a bloom cycle and how this impacts bloom dynamics. Many physiochemical aspects of snow habitat may impact snow algal biology, including temperature, light, and nutrients. Temperature is known to play a role in snow algal blooms generally, again because of the need for liquid water. However, the temperature of snow remains consistently at or around 0°C during blooms, while air temperature can fluctuate (Kvíderová 2010). While many snow algal taxa can survive freeze-thaw cycles, average air temperatures need to be above 0°C to support growth (Hoham 1975a). Novis (2002a ) observed higher growth rates during rainstorms on snow, where melt rates and liquid water availability were highest. Light is also an important factor for all photosynthetic organisms. Snow algae are adapted to high-light environments; They can experience up to 5,000 mol photons m^−2^ s^−1^ with changes in light sometimes triggering life cycle transitions (Hoham and Remias 2020). Lastly, nutrients are known to be important factors in algal blooms. Snow is known as a low-nutrient environment, and the limited studies of nutrients in the snow habitat are not conclusive as to exactly how natural nutrient levels in snow habitat affect snow algal blooms (Hoham and Remias 2020). However, snow algal blooms have been shown to intensify in response to addition of nutrients (Ganey et al. 2017).

To address the dynamics of a snow algal bloom in situ under natural conditions, we studied a known recurrent pink snow algal bloom in the North Cascade Mountains (R. Kodner, pers. obs.). The North Cascade Range is a low-elevation mountain range and is the most glaciated mountain range in the continental US. The range runs north-south through Washington, with high snowpack accumulation and diverse geomorphology. The Cascades are known to host large and diverse snow algal blooms both above and below tree line, many of which were characterized in the 1970s (Hoham 1974a, 1975b, Hoham and Mullet 1977, Hoham and Roemer 1979). We have been observing a recurring bloom of *Chlamydomonas* sp. since 2017 (R. Kodner pers. obs., Matsumoto, et al. in revision) on snow on top of Upper Bagley Lake in the Mt Baker National Forest, WA. The bloom can be well defined in terms spatially, occurring only on the snow-on-lake habitat, and the end of the bloom is determined by the seasonal loss of the snow habitat by mid-summer. This snow algal bloom is an early season bloom (begins with the first spring melts) and is accessible to sampling year-round.

These combined characteristics give the Bagley Lake *Chlainomonas* sp. bloom a natural experimental design to observe the spatial and temporal changes during the bloom cycle from the start of visual surface biomass through the duration of habitable snow in the Bagley Lakes system. Our objective in this study was to characterize *Chlainomonas* sp. bloom dynamics by integrating measurements of environmental conditions (temperature, light) of the habitat, observations of community structure (using microscopy and amplicon metabarcoding), photophysiology, and snow surface color that marked the observable “bloom” on snow surfaces. Through this study design, we were able to address how the microbial community composition, algal physiology, and color intensity of snow surfaces as the bloom progressed over space and time.

## Methods

### Study site

**Figure 1.**
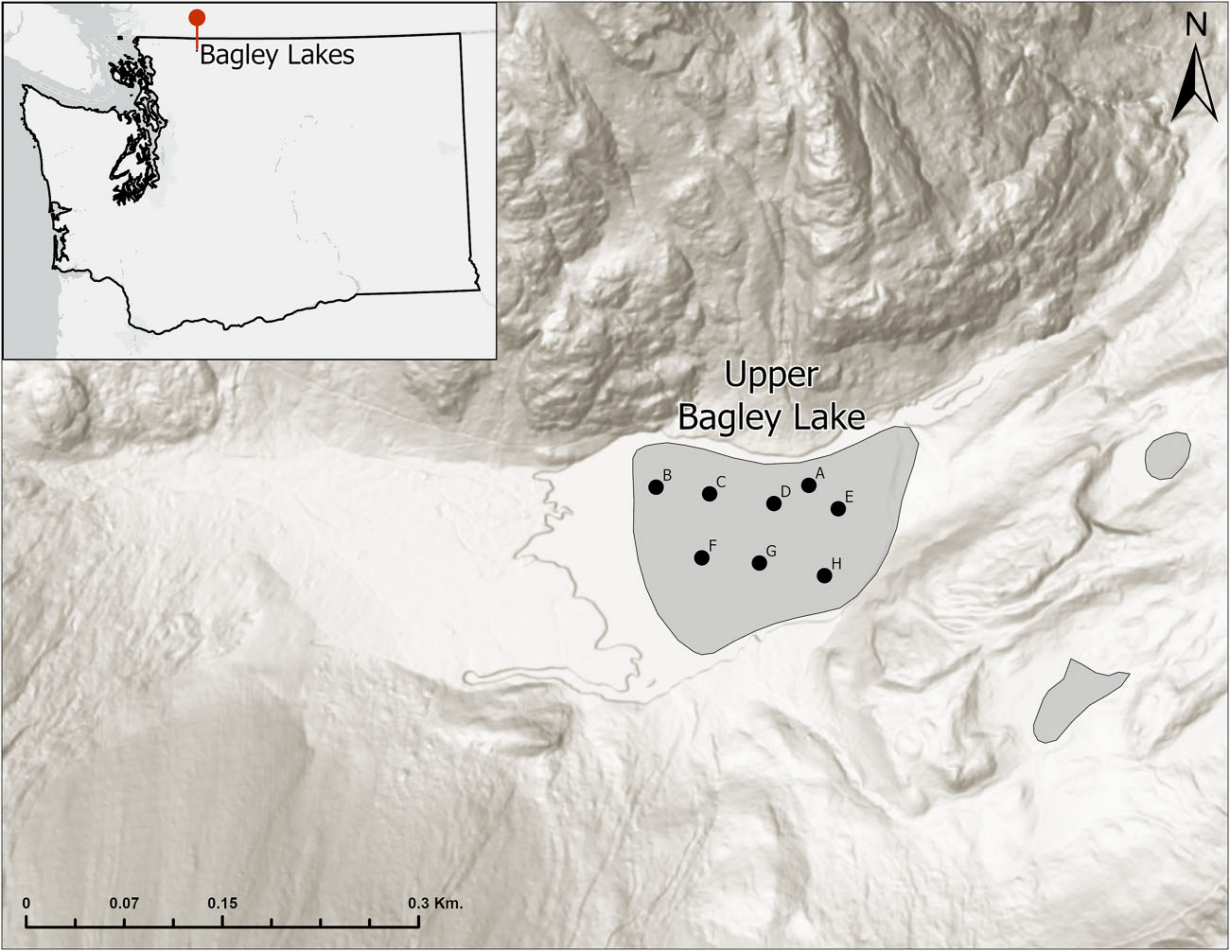
A map showing sampling locations A-H situated on Lower Bagley Lake in the Cascade Range, Washington USA. This map was created in ArcGIS. Inset map shows the broader geographic context for the location of this study.

Upper Bagley Lake is in the North Cascades mountains of western Washington State. The lake (1277 m above sea level) sits 11.8 km northeast of Mt. Baker at 48.8632, −121.6793 (Fig. 1). The lake is situated in a basin that is flanked by Mt Herman to the north and Table Mountains to the west. During the late autum - early summer, Bagley Lake is typically covered in snow and is surrounded by snow covered slopes. This area receives direct sun from early morning through late afternoon. Bagley Lake is unique in that it does not ice over as many alpine lakes do in winter. Instead, fall snowpack begins to accumulate in somewhat mild temperatures that hover around freezing, so that the lake surface itself does not freeze. The lake is also shallow, with depths that range from 2-3 m in the summer. Thus, the lake water is absorbed into the snowpack as it accumulates in the winter, and floats over a thin layer of slush and water at the lake bottom until the spring melt. The snowpack that covers the slush mixture is roughly 0.5 m deep before the slush transition in the spring immediately preceding the bloom and becomes shallower over the spring to summer transition before the snow on the lake melts completely. Even toward the end of the bloom where open water is found on most of the perimeter of the lake, the large residual snow rafts are supportive and can be walked on.

The snow algal blooms in the basin appear first on the snow that covers Upper Bagley Lake and persist on the snow-on-lake habitat until snow completely melts off the lake. Complete snow melt on the lake surface regularly occurs in the middle of July but can be as early as mid-June. The onset of the bloom ranges from late April to late May (R. Kodner, pers. obs.) and continues through mid-summer until the snowpack disappears from the surface of the lake. Snow algae often initially appear at the north side of the lake, suggesting they may be responding to the south facing light environment. Typically, by the end of the bloom on Bagley Lake, snow algae can be seen in the surrounding snow slopes, but not before, suggesting the snow-on-lake habitat is habitable before the snow over rocks or vegetation. We have observed this pattern of early blooms on the lake, which are dominated by *Chlainomonas* sp., every year since 2017. This species is an undescribed species of *Chlainomonas* (Novis et al. 2023, Matsumoto et al. in review). This species represents over 98% of amplicons from lake samples from 2017 to 2019 (R. Kodner, unpublished data) and in all microscopy samples collected from 2017 to 2023 (R. Kodner, unpublished data). In 2021, we identified the start of the *Chlainomonas* sp. bloom by the appearance of pink patches on 20 May and sampled weekly until 9 July.

### Sample collection

To capture the spatial variability of the bloom across the lake, we set up a sampling grid at 60 m intervals across the lake using ArcGIS (Fig. 1). Point A was the first observed instance of snow algae (pink patches) at T_o_ (Week 0) and was found outside the pre-determined grid. Sample locations B-H were then determined from the grid and sampled starting T_1_ when snow algae patches were found across the lake. This grid served as the scaffolding for all measurements and samples across the season. Samples were collected for light and electron microscopy, DNA extraction, and relative chlorophyll fluorescence analysis at each sample location.

### Environmental data

We monitored temperature and light conditions on the bloom from 21 May to 9 July 2021 using three HOBO loggers set across the lake at sample locations A, B, and G. Loggers were mounted flat, facing the sky, on white PVC pipes and took readings every five minutes. As the snowpack melted throughout the bloom, we repositioned the loggers each week to keep them as close to the snow as possible. Additionally, we gathered air temperatures from the surrounding area from the Northwest Avalanche Center (NWAC 2022) station located at Heather Meadows (elevation 1283 m). We summarized maximum and minimum daily temperatures for each day on the bloom as an average of the three loggers (+/− SD). The temperatures from the NWAC station were single-point values with no measure of spread. We summarized the light condition of the bloom as maximum daily light averages for the three loggers. We then averaged the maximum daily light values (n = 3 +/− SD) for the duration of the bloom.

### Microscopy

At each site (n = 8 per week), we collected 15 ml of fresh pink snow from within a 5 m radius of the site pin and stored the samples in an insulated cooler packed with snow to keep cold without freezing. All samples were kept in the dark and remained as snow, and were imaged between 12 and 48 after collection. To image, 20–30 ul of liquid water from the sample that began to melt at ambient temperatures was added to a slide. We took 20 images from each of the eight samples at 400x total magnification (EVOS XL Core Imaging System, Thermo-Fisher, Waltham, MA, USA), and visually assessed the cell structure and morphology. Samples for scanning electron microscopy (SEM) were collected from snow or meltwater. Biomass from samples dried on circular cover slips and coated with gold and palladium and imaged with a Tescan Vega 3 with Oxford EDS (Brno, Czech Republic) SEM.

### DNA extraction and amplicon sequencing

For DNA collections, ∼ 20 ml of snow water equivalent was added to a 50 mL conical vial and mixed into 20 ml RNA buffer preservative like RNAlater (935 ml MilliQ water, 700 g Ammonium sulfate, 25 ml of 1 M Sodium Citrate, and 40 ml of 0.5 M EDTA, adjusted to pH 5.2 and autoclaved). Samples were stored at 4^o^C before being filtered onto 25 mm 0.2 µm Supor filters (Pall, New York, USA). Prior to extraction, samples were washed three times with PBS buffer. Filters were cut with sterile scissors, transferred to bead beating tubes from Zymobiomics kit (Zymogenetics, Seattle, USA) DNA miniprep extractions kits and subjected to bead beating for 5 mins. Extractions proceeded from this step as following with the Zymobiomics DNA miniprep kit instructions.

DNA was quantified using a Nannodrop to ensure extractions were successful. We submitted DNA to the University of Minnesota Genomics Center for amplicon library prep and sequencing of the V9 region of 18S SSU rRNA gene sequence using MiSeq Illumina 2 x 300 bp chemistry. UMGC prepared dual indexed Nextera XT DNA libraries following their improved protocol for library preparation which enables detection of taxonomic groups that often go undetected with existing methods (Gohl et al. 2016). The V9 region of the 18S rRNA was amplified using primers Euk1391F 5′-GTACACACCGCCCGTC-3′ and EukBr 5′-GATCCTTCTGCAGGTTCACCTAC-3′ (Amaral-Zettler et al. 2009, Stoek et al. 2010) and the ITS2 region was amplified using primers Coleman c 5′-GCATCGATGAAGAACGCAGC-3′ and Coleman b 5′-GGGATCCATATGCTTAAGTTCAGCGGGT-3′ (Coleman et al. 1994, Segawa et al. 2018). UMGC prepared dual indexed Nextera XT DNA libraries following their improved protocol for library preparation which enables detection of taxonomic groups that often go undetected with existing methods (Gohl et al. 2016). OTUs were assembled from the V9 reads using mothur following following the MiSeq SOP (Kozich et al. 2013). Amplicon sequence variants (ASVs) were recovered from the ITS2 data using DADA2 (Callahan et al. 2016) following the SOP at https://benjjneb.github.io/dada2/. The top 24 amplicons identified, which represent at least 90% of all sequence reads in the most samples, were then annotated with BLAST using nr from NCBI. Information of reference hits to all algae and chytrid taxa found in Supplement 2.

### Relative chlorophyll fluorescence

We measured the maximum quantum yield of PSII (Fv/Fm) of the bloom each week using an Opti-Sci OS1p field PAM fluorometer with a phytoplankton cuvette (Hudson, NH, USA). We took all readings between 10:00 and 12:00. All samples had similar light histories from the time of collection to measurement. Following the preset sampling grid ((n=8 per week)), we collected 50 ml of pink snow in Falcon Tubes from each site within a 5 m radius of the site marker/GPS waypoint. We then allowed snow to partially melt into a slurry (10-15 minutes), allowing some algal cells to settle to the bottom of the tube. We collected algae from the bottom of the Falcon tube and transferred three 1.8 ml aliquots to the measuring tubes. Samples were loaded into the phytoplankton cuvette and dark adapted for 10 minutes. Following dark adaptation in the aquatic cuvette, we measured the algae's response to light according to the OS1p manual. We adjusted the modulation intensity of the actinic light prior to measurement to achieve a baseline fluorescence (F_t_) high enough for a successful reading. We used a saturation light pulse length of 1.5 s to achieve enough fluorescence for an accurate reading, which differed from previous research (Shreiber 1995). This optimized pulse length gave us a clear fluorescence peak with a flat top compared to a weak and inconsistent peak with shorter saturation durations (Roseqvist and van Kooten 2003).

### Cell density

We calculated algal cell densities of the samples used in PAM fluorescence analysis in weeks 2–6. A 1.8 ml aliquot were collected directly from the measuring tubes and were fixed with 2% paraformaldehyde immediately after fluorescence measurements. Fixed samples were then counted in a hemocytometer chamber using light microscopy (as described above). We quantified cells from ten 1 x 1 mm boxes, from two prepared slides per preserved sample. The average number of cells per ml was calculated from the technical replicates.

### Color intensity of bloom patches

To quantify the intensity of color on the snow each week, five representative patches of algae were selected at each site and photographed for image analysis. To standardize imaging of the snow algal patches, we built an imaging stand out of PVC pipe with camera holder on top of a 0.5 x 0.5 m square quadrat (shown in Supplement 1). The camera was held in a camera holder on top of 1.5 m arms, standardizing the imaging distance and camera orientation. At each selected patch, the PVC quadrat imaging stand was leveled on the snow surface surrounding the patch to create a 0.5 m square border. A digital SLR camera (HD-45002 DSLR mark II Cannon) was placed on the top of the imaging stand with the lens facing directly towards the snow using auto focus mode. We took three replicate images of each patch (n=15 images per site) and best the photo was used for quantification. The imaging stand and observer were positioned to minimize shadows.

We quantified the extent of pink snow coverage in images via analysis of pixel intensity using FIJI-ImageJ (Abràmoff 2004). Each image was processed with the following protocol in ImageJ: We selected a square within the borders of the PVC pipes that included as much interior space as possible. Within the Color Threshold window and with a red threshold, we set the hue between 215 and 255 while saturation and brightness were adjusted to overlay all areas determined to show pink snow in the image. The area covered by the threshold was selected with “select” in the Color Threshold window. We then set the measuring stats using “Set Measurements”. These included: area, min and max gray value, integrated density, and mean gray value. We measured the area with “measure” to give us the values for the selected pixel area. We then duplicated the selected square for each photo to include only snow within the quadrat perimeter. This approach ensured the color threshold analysis was performed on the same square of snow within the PVC quadrat for each image. This method also eliminated measuring any snow outside the quadrat area. All area values from quadrats from individual site were then averaged, giving us eight individual final values per week. We used the integrated density values as the best measure of color intensity. These intensity values are scale-independent and unitless, and comparable across all images in the project. The individual picture scaling allowed us to compare values among pictures without the need for external comparisons to other bloom situations.

### Qualitative assessment of bloom patchiness across the lake (visual survey)

We assessed the extent and intensity of the algal bloom across Bagley Lake each week with a systematic visual survey of the sampling area using a team of observers. These observations were taken with the naked eye, and estimates of intensity was calibrated to standardize observations among observers each week. Using the established the sampling points (B-H) that marked a 6x6 120m^2^ grids (Fig. 1), we divided each internal square into a grid that was two to three squares wide and 10 squares (10 m each) long, giving us 20–30 pixels. Two to three observers simultaneously moved in parallel across the bloom in either north or south directions. After 10 m, the observers stopped and quantified the intensity of the bloom using a 0–4 scale (0 = none, 1 = rare, 2 = sparse, 3 = common, 4 = abundant). Individual observers calibrated their assessment of color scoring after the first pass. An independent observer coordinated the movement of observer team and observations and recorded data. After recording a spot, all observers repeated the 10 m progression. We omitted observations when we encountered either debris or surface water/open water that made the visible presence of algae impossible, thus the number of observations were reduced each week as the snow on the margins of the lake melted. No observations were made during week 3 due to snowfall the night before (thus difficult to observe the patches) or during week 8 due to degradation of the snowpack. We then transposed the observations into Excel to create heat maps using conditional formatting for color gradation (0–4) for cells that matched the observation grid. Darker colors represent more intense algal presence. Black squares on the map indicate that no measurement was taken for that square.

### Statistical analyses

We ran all statistical analyses in R ver. 3.3.0 (R Core Team 2022). We ensured that the assumptions of normality and equal variance were met prior to statistical testing. If data did not initially meet the necessary assumptions, we used log-transformations. Unless otherwise stated, all tests used an α = 0.05 to determine the presence of significant patterns. We quantified differences in Fv/Fm values of the snow algal biomass among observation weeks using a one-way ANOVA with week as a fixed factor (n = 8 per week). We compared the visual density of snow algae among weeks on log-transformed values with a one-way ANOVA with week as the fixed factor (n = 8 per week). Similarly, we compared the relative proportion of *Chlainomonas* sp. in the microbial assemblage in 18S SSU rRNA amplicon sequence data with a one-way ANOVA among weeks. Week 0 was kept in the graphical summary as a reference for the population at the onset of the bloom. Finally, we compared cell counts of samples used for fluorescence analysis with a one-way ANOVA on log-transformed cell counts (n = 8 per week). Any ANOVA that was found to be significantly different (*P* < 0.05) was followed by a Tukey *post*-*hoc* test to determine pairwise differences among the treatment levels. A correlation test was used to assess the relationship between Fv/Fm and integrated density where data was available from both measurements (n=6).

## Results

### Environmental conditions

**Figure 2.**
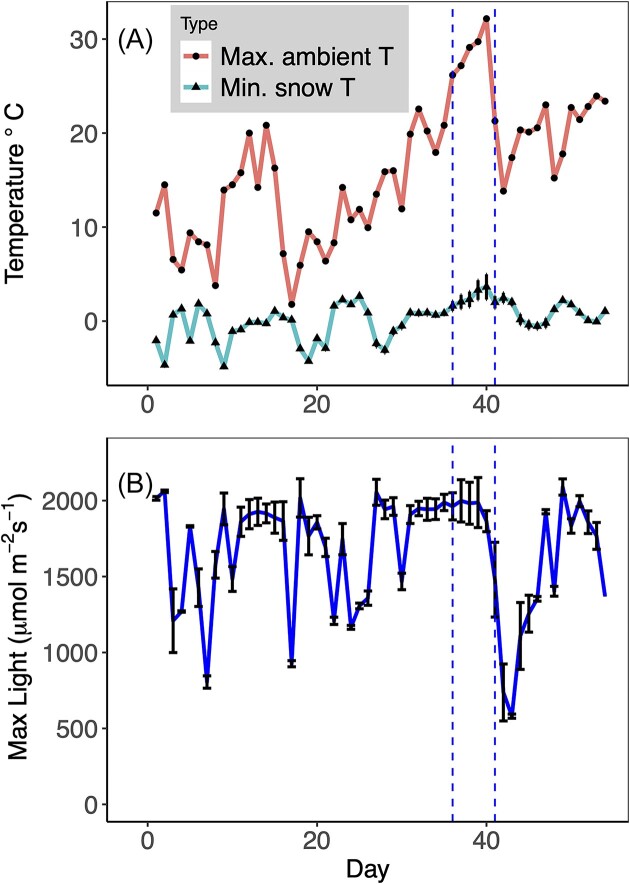
Environmental Data (temperature and light) measured across Bagley Lake in 2021. Snow surface temperature and light recorded by 3 Hobo loggers placed across the study site (1310 m) elevation and averaged. Ambient air temperature recorded by Northwest Avalanche Center telemetry station at Heather Meadows (1280 m elevation) located approx. 1 km from center of Upper Bagley Lake. **A**) Daily average (n = 3, means +/− SE) surface snow temperatures (minimum (blue) ambient maximum ambient air temperature (red)) for the area. **B**) Maximum daily light intensity (μmol m^−2^ s^−1^) (n = 3, means +/− SE). Vertical blue lines show the location of the atmospheric heat dome that occurred during week 6.

Minimum average daily snow surface temperatures ranged from −4.8 to 3.6^o^C while atmospheric temperatures ranged from 1.7 to 29.7^o^C. Temperatures measured on the bloom were consistently lower than atmospheric temperatures at Heather Meadows (Fig. 2A) but had similar patterns temporally. There was little daily variation in temperature among the three corners of the study area. Washington State experienced a heat dome during the last week of June 2021. This coincided with the sixth week of bloom monitoring at Bagley Lake. During that time, both ambient and snow-surface temperatures increased with a record ambient temperature of daytime high of 34^o^C and nighttime low of 26.5^o^C measured at the NWAC weather station. Maximum average daily light intensity (LUX) experienced by the bloom fluctuated between 45 and 120 x10^3^ LUX (Fig. 2B). Vertical lines in Fig. 2 indicate the start and end of the atmospheric heat dome event in northwest Washington in late June 2021 (Emberton et al. 2022).

### Light microscopy

**Figure 3.**
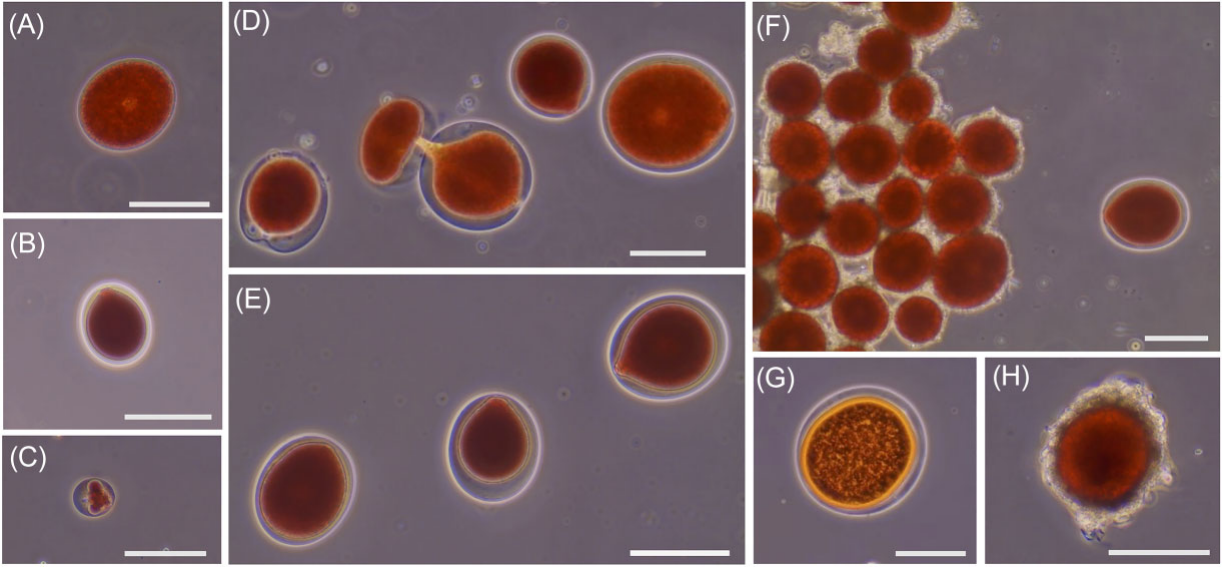
Representative *Chlainomonas sp*. cell morphologies found throughout the 2021 bloom, all of which have been observed as part of the life cycle (Matsumoto et al in review). All scale bars are 60 μm. **A**) Large *Chlaionomonas* sp. cell with thin cell wall and visible central nucleus 05/20/21 (Week 0) first visible patches of the bloom; **B**) Common non-motile vegetative *Chlaionomonas* sp. cell with thick cell wall 05/20/21 (Week 0) first visible patches of the bloom; **C**) Small *Chlaionomonas* sp. cell morphology with condensed cell contents 05/25/21 (Week 1) from site C; **D**) Image of 3 *Chlaionomonas* sp. cells with morphology and size variation co-exiting along with a cell undergoing the unique cell division from a single patch collected on 07/06/21 (Week 7) from site E; **E**) Three common *Chlaionomonas* sp. morphologies co-existing in a patch collected on 06/16/21 (Week 4) from site G; **F**) Rafted *Chlaionomonas* sp. spore morphologies co-existing with the common vegetative cell morphology in a single patch collected on 06/22/21 (Week 5) from site D; **G**) The rare *Chlaionomonas* sp. morphology with dense cell contents and think orange layer around cytoplasm collected on 07/06/21 (Week 7) from site E; **H**) A single *Chlaionomonas* sp. spore morphology showing thickened cell wall collected on 07/13/21 (Week 8) from site C.

All pink snow samples that were imaged were dominated by *Chlainomonas* cells. *Chlainomonas* species display morphological variability across life stages (Hoham 1974a, Hoham 1974b, Novis 2002b, Novis et al. 2008, Remias et al. 2016, Procházková et al. 2018, Matusumoto et al. in review). All species from this genus have a set of distinguished from other snow algae species with the large ellipsoidal to nearly spherical shaped vegetative cells and they possessed a papilla (thickened cell walls with flagellar openings at the anterior pole) and a pseudo-papilla (thickened posterior cell wall). This morphology and the variation in size, cell wall, and papilla or pseudo-papilla are see in Fig. 3A–F. The *Chlainomonas* species that dominates Upper Bagley Lake is a new species and is larger than *Chlainomonas rubra*, though it nests within other sequences identified as *C. rubra* (Novis et al. 2023, Matsumoto et al. in review). *Chlainomonas* sp. common cell morphologies observed during the 2021 are similar to other described species of *Chlainomonas* and the common morphologies (Fig. 3A-E), including the unique cytoplasm extrusion, described by others as cell division (Novis et al. 2008) seen in the middle cell in 3D. Morphologies show in Fig. 3A–E were observed throughout the bloom and across the lake. Less common cells types, including Fig. 3G and was seen in weeks 3–6, and Fig. 3C which was more seen in the early weeks of the bloom. During and immediately after the heat wave event in week 6, we observed cells with spore-like walls clumping into rafts (Fig. 3F). A spore morphology is shown in Fig. 3F, in relation to vegetative cells and Fig. 3H (see Supplement 2 for SEM image of spores).

**Figure 4.**
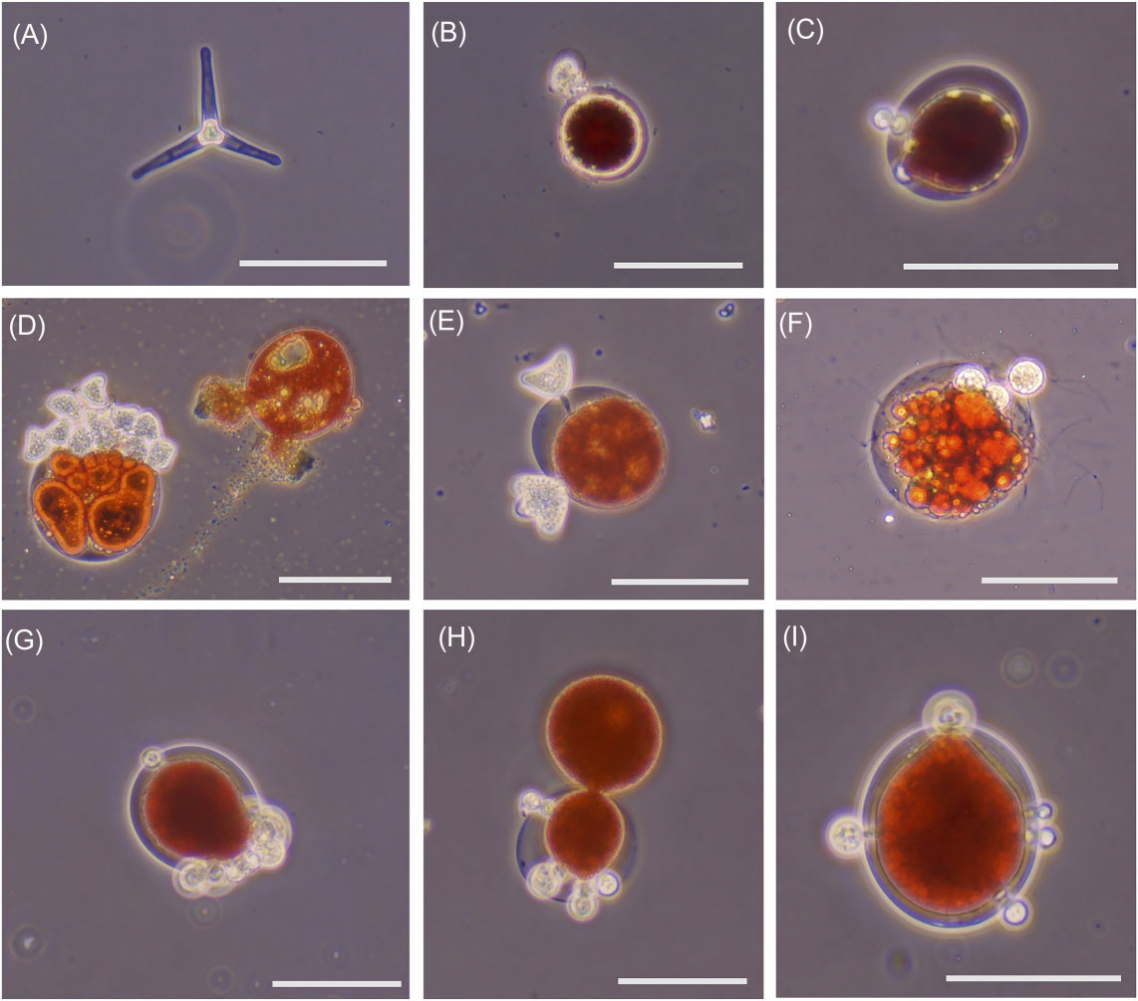
Morphology of fungi/chytrid infections during bloom. **A**) *Chionaster* sp. collected on 052521 (Week 1) from site D.; **B**) Small infected *Chlainomonas* sp. cell with round fruiting body collected on 05/25/21 (Week 1) from site A; **C**) Larger infected *Chlainomonas* sp. with parietal green spots collected on 06/16/21 (Week 4) from Site H. Small round fruiting bodies penetrating cell wall; **D**) Severely infected *Chlaionomonas* sp. cell with multiple triangular fruiting bodies collected on 06/08/21 (Week 3) from Site D (left) and lysing cell (right)​; **E**) Infected *Chlaionomonas* sp. cell showing triangular fruiting bodies penetrating cell wall, collected on 06/16/21 (Week 4) from Site F; **F**) Infected *Chlaionomonas* sp. cell showing hyphae and fruiting body collected on 06/08/21 (Week 2) from site A; **G**) Infected *Chlaionomonas* sp. cell showing small round fruiting bodies on 06/29/21 (Week 6) from Site C ; **H**) Infected *Chlaionomonas* sp. undergoing unique cell division with parent cell showing small round fruiting bodies collected on 06/29/21 (Week 6) from Site G; **I**) Infected *Chlaionomonas* sp. showing multiple round fruiting bodies penetrating cell wall collected on 07/06/21 (Week 7) from site C.

Infection of the algal cells by a chytrid fungus was also frequently observed (Fig. 4). Infected cells were seen in every week of the bloom, but the most infected areas of the lake were not consistent over time. in addition, the fungus *Chionaster nivalis* (Fig. 4A) was present in each week of the bloom. Two distinct Infection morphologies were observed (round and triangular) and often several fruiting bodies can be seen on cell surfaces (Fig. 4B–I). In some cases, the infection can be seen penetrating the cell wall of the algae (Fig. 3E–I). Fungal or chytrid hyphae can also sometimes be seen (Fig. 3H). On several occasions, we observed lysing events, particularly with larger cells once under the microscope (Fig. 4B). Lysing is distinct from *Chlainomonas*’s unique cell division where cytoplasm is extruded out of a vegetative cell to produce a new cell enclosed in a cell membrane (Fig. 3D middle cell) (Novis et al. 2008, Matsumoto et al. in revision). This cell division was observed in weeks 3–6 and was observed in an infected cell (Fig. 4F) suggesting that infection does not prohibit cell division.

### Amplicons for community composition

**Figure 5.**
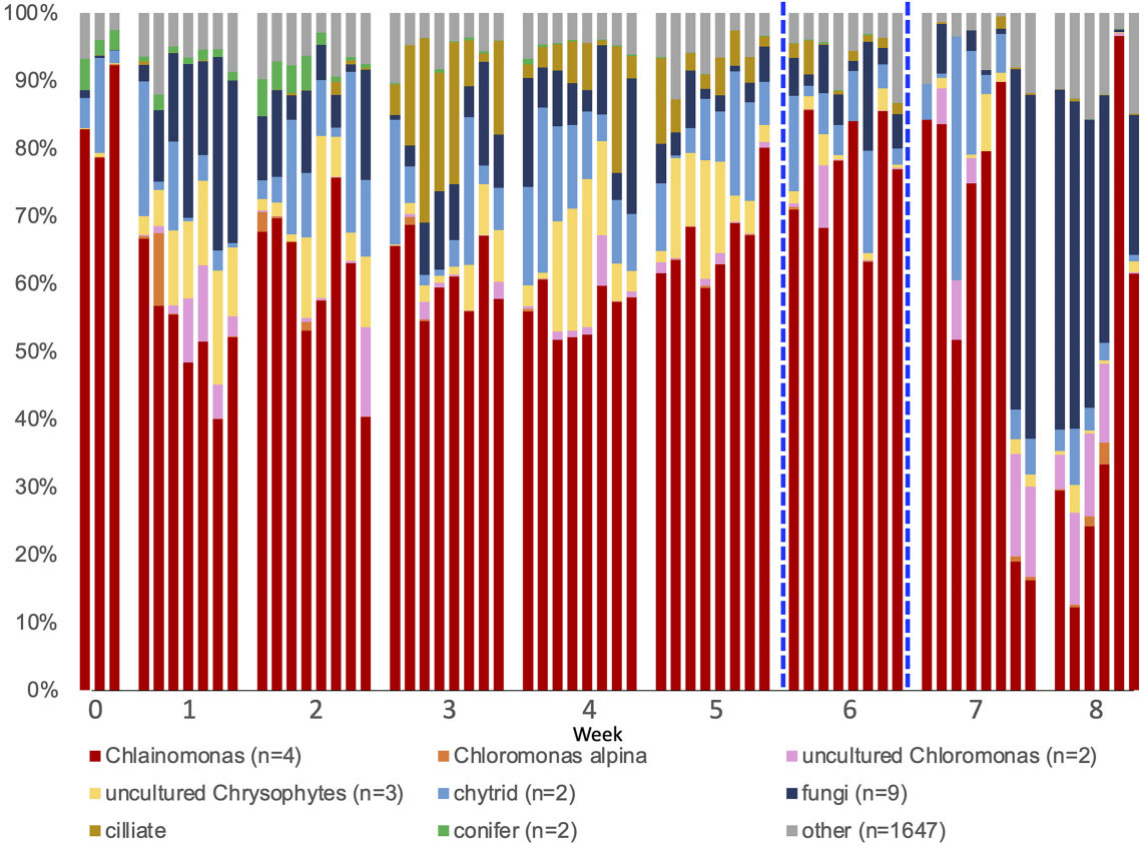
18S V9 amplicon data from each sample, by week. ASVs assigned to the same taxonomic group are binned and the n is of ASV summed in that group. If no n is listed, only a single ASV contributed group. Vertical blue lines show the location of the atmospheric heat dome that occurred during week 6.

Amplicon data from the V9 18S SSU rRNA gene sequence was used to assess total eukaryote composition from samples collected across the bloom. Data are available via NCBI BioProject PRJNA985372. V9 18S SSU rRNA gene Amplicon data show that all samples are dominated by *Chlainomonas* sp. (red taxon, Fig. 5) except for a few weeks toward the end of the bloom when samples became fungal dominated. There are 4 unique amplicon sequence variants that have a best match to *Chlainomonas* sp., but the overwhelmingly dominant ASV (97.3% of all ASVs annotated to *Chlainomonas*) has a 100% identity to full length *Chlainomonas* sp. that appears to be a new species in comparison to the described *Chlainomonas* species described from New Zealand, Japan, and Europe (Matsumoto, et al., in review). All 4 amplicon sequence variants have the same BLAST hit results for reference sequences (Supplement 3). We used the ITS2 amplicons to assess more specific diversity within the dominant *Chlainomonas* clade across the bloom. We recovered only a single ITS2 sequence (OP297764.1) that was annotated as *Chlainomonas* sp. across all samples. This sequence also supports the identification of the Bagley *Chlaionmomons* as a new species (Novis et al. 2023). The relative proportion of *Chlainomonas* sp. measured with the V9 amplicon was constant over the bloom period until the final week where the proportion was significantly higher (F_5,41_ = 9.49, *P* < 0.001) (Fig. 5).

Patterns in other taxa can be also seen over the course of the bloom. Conifer DNA appears in the data early in the bloom season, ciliates are found mid-bloom, where populations may grow in response to increase algal biomass. Chytrid and fungal taxa (ASVs binned by functional groups) remain relatively stable through the bloom, except for some fungal dominated samples at the end of the bloom, but this was not consistent in every sample. Chytrid infection was observed thought the bloom in the microscopy data (Fig. 4) and amplicons are present through the entirety of the bloom as well. Replicate samples are shown to indicate patch level variability across the bloom. Each of the two abundant chytrid amplicon sequence variants hit different sets of reference sequences. The dominant ASV has a 100% match to an environmental sequence from the Swiss Alps (accession AJ867631.1) and the next most abundant chytrid sequence's best match to a reference is only at 98.4%. Uncultured Chrysophytes (likely a single taxon represented by 3 ASVs) were also common in the amplicon samples in weeks 1-5, though were not observed commonly in microscopy samples. Chrysophytes likely have a higher DNA extraction efficiency from mixed community samples than *Chlainomonas sp*. that has an extremely thick cell wall that is hard to break in single cell PCR attempts and is difficult to penetrate with DAPI, a nuclear stain that is often easily penetrates cell walls (unpublished). This taxon has been observed in other years and can produce localized yellowish-brown blooms on Upper Bagely Lake, but did not bloom in 2021. Ciliates and Chrysophytes, and to some extent Chytrids, had a reduction in relative proportion during and following the heat dome in week 6.

### Chlorophyll fluorescence measurements

**Figure 6.**
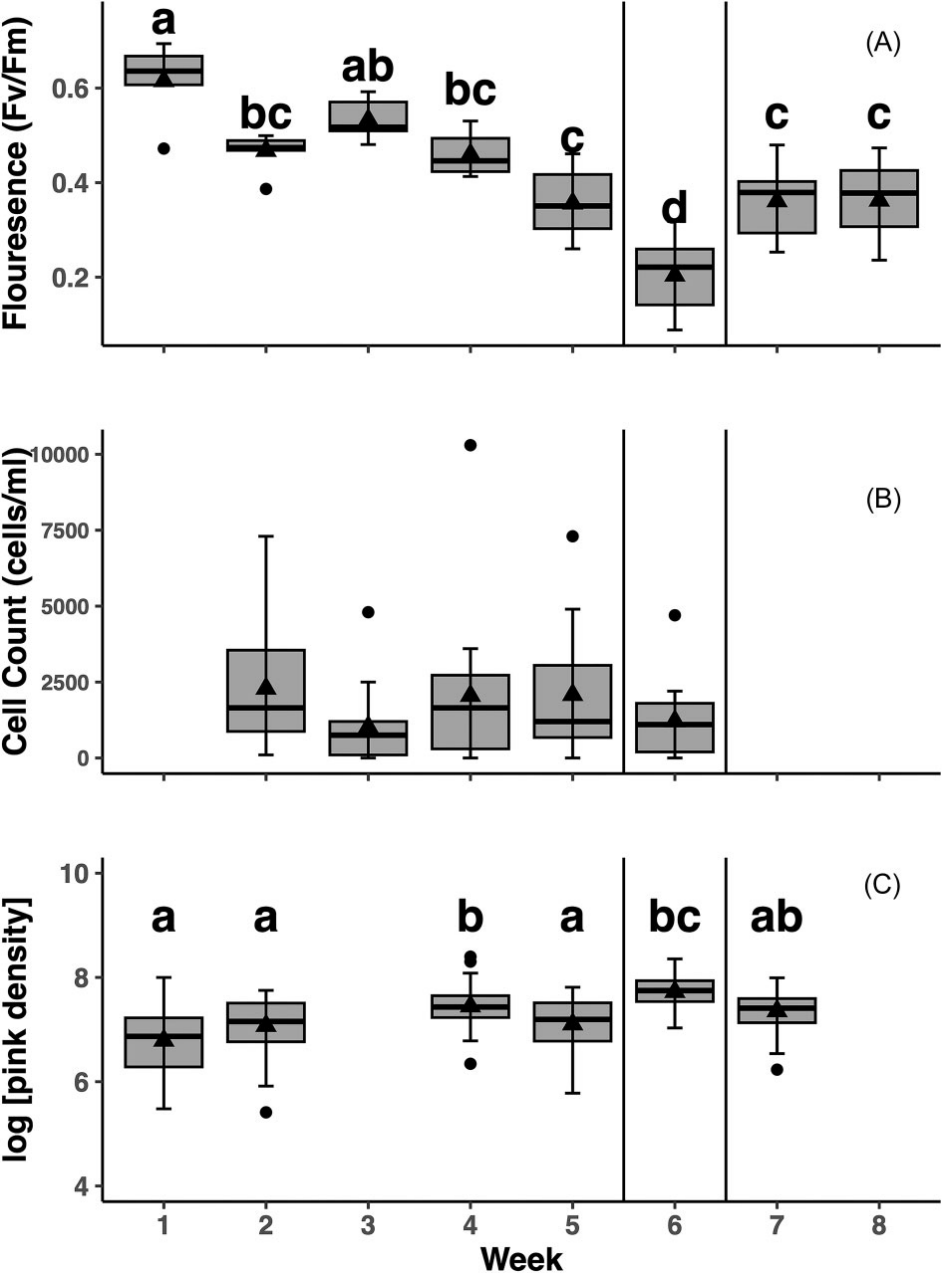
**A**) Maximal photochemical efficiency of PSII (Fv/Fm). Measurements taken each week of 8 weeks. **B**) Cell counts of snow used to measure chlorophyll fluorescence (cells/ml). Measurements taken weeks 2–6. **C**) log-transformed integrated density of color of pink patches on surface snow. Measurements taken weeks 1–2, and 4–7. Week 3 was omitted due to recent snow fall that obscured surface patches. Letters represent significant differences among weeks in each panel determined with an ANOVA and Tukey post-hoc analysis (*P* < 0.05). Vertical black lines show the location of the atmospheric heat dome that occurred during week 6.

The mean Fv/Fm of the snow algal community in the Bagley Lake bloom ranged from a high of 0.62 during week one to a low of 0.20 during week 6 (Fig. 6A). The Fv/Fm values significantly decreased across weeks during the 2021 bloom (F_7,45_ = 22.63, *P* < 0.001) (Fig. 6A). Fv/Fm (or the maximum quantum yield of PSII) was highest during the first and third weeks of the study (Tukey HSD, *P* <0.05) and is consistent with the highest measurements for Fv/Fm that we measure for snow algae that we see in this region (Kodner unpublished data). Fv/Fm then declined to a plateau during week 4 of the study to value that indicate continued photosynthetic activity in the algae but some level of stress in PSII. This plateau was consistent through to the end of the study except for week 6, where we saw lower fluorescence values drop to their lowest levels (Tukey HSD, *P* < 0.001).

### Intensity of bloom patches over time (image quantification and cell counts)

The cell density of algal samples collected from the same sampled used for relative chlorophyll fluorescence measurements were similar among weeks 2–6 (Fig. 6B). Cell densities ranged from 200 to 10 300 cells ml^−1^. Algal patch color intensity on the snow surface (reported as integrated density) increased significantly from beginning of bloom to the end (F_5,41_ = 5.38, *P* < 0.001) (Fig. 6C). Integrated algal density in week 6 was significantly higher than weeks 1, two and five (Tukey HSD, *P* < 0.05), coinciding with decreases in Fv/Fm that week, but not a decrease in cell density. The log of the Integrated density and Fv/Fm is are negatively correlated (with a ρ =−.91, *P* value=.011) suggesting a significant inverse relationship between these measurements during this bloom. However, this analysis included only a small number of measurements (n=6) and is suggestive rather than demonstrative.

### Visual survey of bloom patchiness across the lake

**Figure 7.**
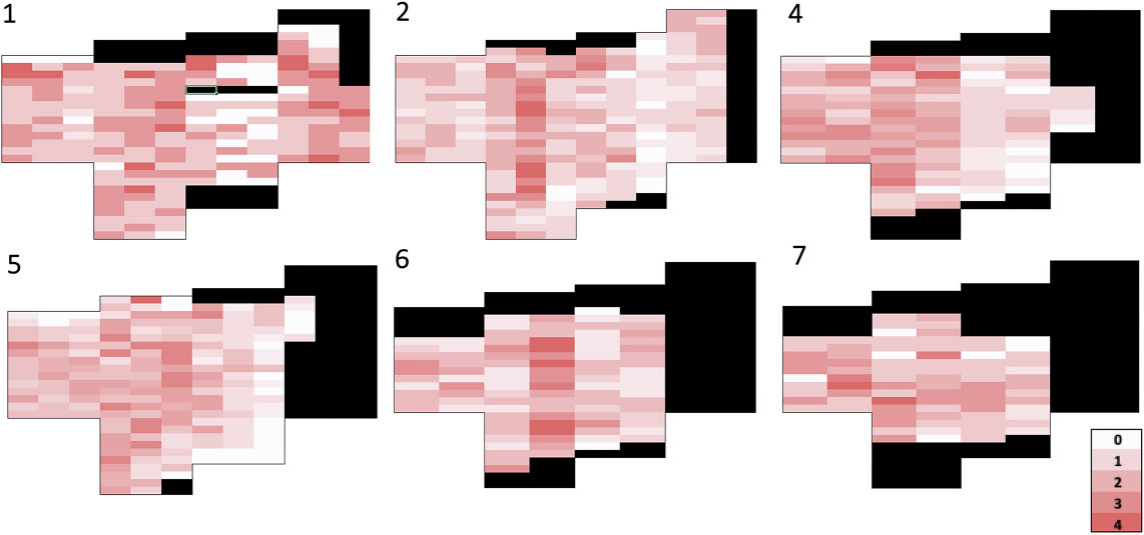
Heat map showing bloom intensity across Bagley Lake, Washington State in the spring and summer of 2021. Numbers in the upper-left corner of each panel correspond to sampling weeks. We omitted week 3 from the survey due to snowfall the day before that obscured the bloom. Bloom intensities ranged from 0 to 4. Blacked out areas were omitted from the survey due to either obstructions or lack of snow in later weeks. No survey was done during week eight because the bloom had degraded and could not be traversed.

The observational transect survey of color across the survey area each week throughout the bloom showed heterogeneity with no clear visual progression among weeks (Fig. 7). We observed patch intensities from 0 (no color) to 4 (darkest pink) in all survey weeks, across the study area. The intensity of a given area shifted weekly within each sampling area in the survey grid as well as across the whole study area. The snow melted on the lake surface as the season progressed, leading to a smaller overall area available for algae to grow and thus a smaller survey area each week. This loss of habitat was on the east side of the lake, with additional losses along the north side (Figures 1 and  7).

## Discussion

This study aimed to understand the spatial and temporal dynamics of a recurring alpine snow algal bloom. We characterized this bloom weekly over the growing season on Bagley Lake, which lasted for eight weeks in the spring of 2021. We found that the bloom developed in patches across the lake area and snow algal cell abundance to be variable but on average, constant across the season with no spatial or temporal trends. Despite the consistency in cell abundance, the color intensity of individual bloom patches generally increased throughout the bloom with a heterogeneous patchy distribution that also had no spatial pattern. *Chlainomonas sp. *consistently dominated the eukaryotic community during the bloom where cell types were mixed throughout the patches. This suggests that life cycle development does not appear synchronized spatially or temporally either. However, the Fv/Fm of communities steadily decreased throughout the bloom season, suggesting some level of community-level stress that is independent of cell abundance or life-cycle but is inversely related to color intensity, which is a measure of bloom intensity on snow surfaces. The patterns we observed of *Chlainomonas* sp. blooms on Upper Bagley Lake indicate that, at least for this system, the broad-scale bloom dynamics are starkly different from those in well studied in marine environments (Townsend et al. 1994; Strom et al. 2001; Svedrup 1953), where we would see exponential growth followed by decline and synchronized germination.

### Environmental sources of stress

Algae in this system could be experiencing stress from several sources including environmental stresses and biological stresses like infection or competition. Snow algae experience environmental stress from multiple physio-chemical parameters, including water, temperature, light, and nutrients. The impact that environmental conditions like light and temperature have on the photosynthetic capacity and growth of snow algae may directly impact the longevity of the snowpack environment. Higher average Fv/Fm values at the beginning of the bloom suggest that the *Chlainomonas* sp. was experiencing more optimal conditions and thus less stress than at the end of the bloom. While light and temperature are central to photosynthetic processes (Remias et al. 2005, Stibal et al. 2007), it is overly simplistic to think they alone drive production. A combination of light, temperature may have supported optimal conditions, and thus adequate snow water equivalency (Hoham and Remias 2020, Remais et al. 2016), as well as available nutrients (Jones 1991, Hoham and Ling, 2000). We see a decrease in Fv/Fm during the heat dome at the end of June (Fig. 2). While daily maximum temperatures on the snow surface remained consistent during this study as measured with environmental sensors on snow surface, ambient air temperatures in the region spiked over a 7-day period, coinciding with significantly lower average Fv/Fm measured from in situ samples measured during this heat even (Fig. 6A). This dip indicates the algae were experiencing stress, though the exact mechanism for the stress is unclear. The snow surface temperature should be near 0°C in all melting snow environments. However, the snow temperature can increase above 0°C and even small fluctuations could cause stress. We have measured snow temperatures of +0.2–0.3°C in the snow on Upper Bagley Lake on a more “typical” warm day with air temperatures ranging from 12 to 18°C (unpublished), so it's possible that temperatures up to 34°C raised the snow water temperature enough to cause increased stress though measures of snow water temperatures with a precision of a tenth of a degree were not available in this study. Chlainomonas cells are known to be very sensitive to warming, and flagellate cells can loose their flagella immediately upon warming under the microscope, which is another indicator of stress (Hoham 1974a, Kodner pers. obs.).

Near-freezing environments like snowpack are limited by liquid water (Hodson et al. 2008, Anesio and Laybourn-Parry 2012), and *Chlainomonas* species prefer a wetter environment than some other snow algae (Remais et al. 2016, Novis 2002a, Novis et al. 2008) including very wet snow-on-lake (slushy) habitats (Procházková et al. 2018, Remias 2011). Snow water equivalence of the snowpack supplies the aquatic medium in which snow algae live (Jones et al. 2001, Kuhn 2001). It is in this ephemeral water that the algae grow and reproduce. Interestingly, as a bloom increases in intensity, the dark color on the snow surface reduces albedo, thereby leading to a wetter snow habitat, as positive feedback. Novis (2002a) observed *Chlainomonas kolii* communities increasing in cell abundance in response to rain on snow events, which increased the water content of the snow but would also deliver nutrients.

We did not see overall increases in cell abundance though we did see an increase in snow color intensity in 2021. It can be challenging to quantify the abundance of algae in snow, especially in patchy blooms where density will be dependent on where algae are collected and how much snow is collected across heterogeneous patches that can vary in color and thus cell density, on millimeter scales. Observed increases in cell density or snow color intensity can be a function of population growth or snow ablation and cell concentration. Our findings suggest increases in color intensity are due to concentration and not increases in overall population. The Upper Bagley Lake bloom in 2021 experienced only one precipitation event (snow in week 2) and generally sunny and warm conditions, compared to typical Pacific Northwest spring weather in which Bagley Lakes area experiences regular rain on snow events.

We could not sample for nutrients through the 2021 bloom, because COVID-19 restrictions impacted our study design and implementation. Nutrients are often very low in snow systems. Oligotrophic snow conditions can progressively become more extreme across the bloom season — one half of spring snow nutrients are estimated to flush out the ecosystem in the first 30% of snow melt (Johannssen and Henriksen 1978). While early-season nutrient conditions may support the onset of the Upper Bagley Lake bloom, increasingly oligotrophic conditions possibly limit growth and stress algae, leading to low maximum quantum yield of PSII. Changes in life stage, either on the macro- or microscale, are associated with different nutritional requirements (Procházková et al. 2020, Hoham and Ling 2000). Vegetative cells often have different nutritional requirements from reproductive and dormant cells. The temporal heterogeneity in morphology of *Chlainomonas sp*. in 2021 bloom suggests many small populations progressing through growth and development stages throughout the bloom and nutrient dynamics happen on a small scale in patchy blooms. Novis (2002a) observed a decrease in NH4-N over the course of a *Chlainomonas kolii *bloom, and a change in life stage from collard cells early to uncollared cells later in the bloom.

### Biological sources of stress

There are also biological sources of potential stress to the algae and control of bloom dynamics. Because *Chlainomonas* sp. is the dominant taxon and overwhelming the dominant algal taxon, competition with other algal species in not an issue, as it often is in aquatic systems. A major source of biological stress may be infection. Chytrid fungal infections of algal cells were visible throughout the bloom, including on the first day of sample collection when only a few small pink patches were visible on the snow surface. Chytrids are known to occur in arctic, alpine, and glacial environments in heterogeneous patterns (Brown et al. 2015, Kobayashi et al. 2023) and likely rely on snow algal cells for nutritional support. The presence of chytrid fungal cells in phytoplankton blooms can be present in epidemic proportions, however, the extent of infection within populations depends on a suite of environmental conditions (Ibelings et al*. *2004). We observe chytrids in the population through the duration of the bloom. Thus, it is unclear if and how these infections may relate to temporal patterns we observed in algal stress, as measured by photophysiology.

Many of these snow chytrids are novel species (Naff et al. 2013). The dominant chytrid ASV in this study was also observed in an unpublished study of snow from a lake in the Swiss Alps (GenBank Accession AJ867631.1) suggesting that this sequence was derived from white snow, not pink snow. Tucker and Brown (2022) also found high abundances of chytrids in white snow, suggesting these organisms also persist in white snow, and their populations may not be dependent on algal blooms. That said, we commonly observed chytrid cells attached to *Chlainomonas* sp. cells in Upper Bagley Lake . The snow microbiomes in the British Columbia coast range, just north of the Bagley area, also found an infected *Chlainomonas* cell (Yakimovich et al. 2020). So, chytrid infections in this taxon may be common in the Pacific Northwest and/in the wet snowpack habitat It has also been shown the chytrids can infect *S. nivaloides* (Tucker and Brown 2022) and so the relationship may be a common and perhaps a fundamental aspect of algal blooms. Tucker and Brown (2022) did see a higher abundance of chytrids in Cascades samples than in the Rockies, also possibly suggesting a relationship with wet snow.

It is not clear if life cycle dynamics do played a role in the changes in seen in Fv/Fm. *Chlainomonas *spp. have complex life cycles, although the life cycle progression is not fully characterized for any species in this genus (Novis et al. 2008, Procházková et al. 2018, Remias et al. 2016). Because no species from this genus have been successfully cultured, connecting life stages and cell morphologies into a life cycle has been challenging. Like other studies, we observed a heterogenous mixture of *Chlainomonas *sp. cell morphologies throughout the bloom. Surprisingly, despite unsynchronized life cycle development, there was a significant temporal decrease in Fv/Fm in aggregate from bloom samples across the lake. This suggests that the entire *Chlainomonas* sp. community across the lake experiences similar stress that caused the decrease in photosynthetic capacity, regardless of whether the cells are vegetative, dividing, or encysted.

### Bloom origination

Upper Bagley Lake, unlike other alpine lakes, does not fully freeze over in winter, with ice on the surface and liquid water underneath. Instead, the snowpack extends to the lake bottom where it is slushy. One hypothesis for bloom origination is that the algae colonize the snow surface from the snow/water slush underneath the snowpack by swimming in flagellated stages (Hoham and Ling 2000). Supporting this hypothesis, we found *Chlainomonas* sp. spores in lake sediments (Matsumoto et al. in revision). If these algae are swimming up through the snowpack throughout the growing seasons, pinker snow later in the bloom could result from reproduction on snow surfaces and or continued vertical migration of cells. However, increased snow ablation as the season progresses (Jones 1991, Kuhn 2001) would also concentrate cells on the snow surface, leading to more intense coloration on the snow. In this case, ablation alone would not account for the continued patchiness of the bloom, which changes over time, and the unpredictability of the appearence of new patches. A second bloom origination hypothesis is that *Chlainomonas *sp. cells are airborne and deposited on the snow's surface (Smith 2011). Airborne deposition of algal cells would be a radom process, thus also leading to the patchy distribution of the bloom. The proportional consistency of *Chlainomonas *present in the 18S SSU rRNA community sequences suggests the source of all microbial depositions is the same, or that the surface environment selects for a consistent microbial community across the lake. This consistency leads us to believe that the microbial community populating the snow surface on Bagley Lake originates from the same source population, whether that be from airborne deposition or vertical migration from the lake bottom. This question is best addressed with population-level analyses using appropriate genetic tools. Our ITS2 data produced only a single *Chlainomonas* sp. sequence variant (Novis et al. 2023), so if there is population-level diversity in this bloom, it is not resolved at the level of ITS2. We would need more polymorphic markers, such as microsatellites or single nucleotide polymorphisms to determine population sources.

## Conclusions

Our results characterize a geographically defined snow algal bloom, dominated by *Chlainomonas* sp., across a seasonal cycle. We found that during blooms of *Chlainomonas* sp. in Upper Bagley Lake, life cycle is not synced across patches. In contrast, the bloom did show a clear decreasing trend in photophysiology (Fv/Fm) that suggested algae were stressed as the season progressed and responded to an excessive heat event. Additionally, the eukaryote community composition progressively changes over the bloom, suggesting ecological interactions between trophic levels and between algae and their parasites may be influenced by environmental stress like temperature. As our climate continues to warm, low-elevation snow ecosystems like Upper Bagley Lake will become more transient, reducing the growing season for these algae, which may impact the bloom dynamics and the phenology. In addition, extreme heat events likely to be more common, leading to increased photophysiological stress and unknown impacts on life cycle and may impact the ability of this species to bloom in the future. Systems like Upper Bagley Lake, where the bloom is dominated by one species and the start and end of a bloom can be monitored, provide an opportunity to study the complex factors that affect snow algae bloom events in a natural habitat. Further development of these types of studies will allow us to understand the mechanistic drivers of stress in these ecosystems and help predict how these taxa will respond to future warming.

## Supplementary Material

fiad106_Supplemental_Files
