# Supplementary material for: Patchy and Pink: Dynamics of a Chlainomonas sp. (Chlamydomonadales, chlorophyta) algal bloom on Bagley Lake, North Cascades, WA
Source: FEMS Microbiol Ecol. 2023 Sep 7;99(11):fiad106. doi: 10.1093/femsec/fiad106 (PMC10580270; doi:10.1093/femsec/fiad106)
Supplement: fiad106_Supplemental_Files [file fiad106_supplemental_files.zip › Supp_data Supplement_2_SEM.pdf]

Supplement 2

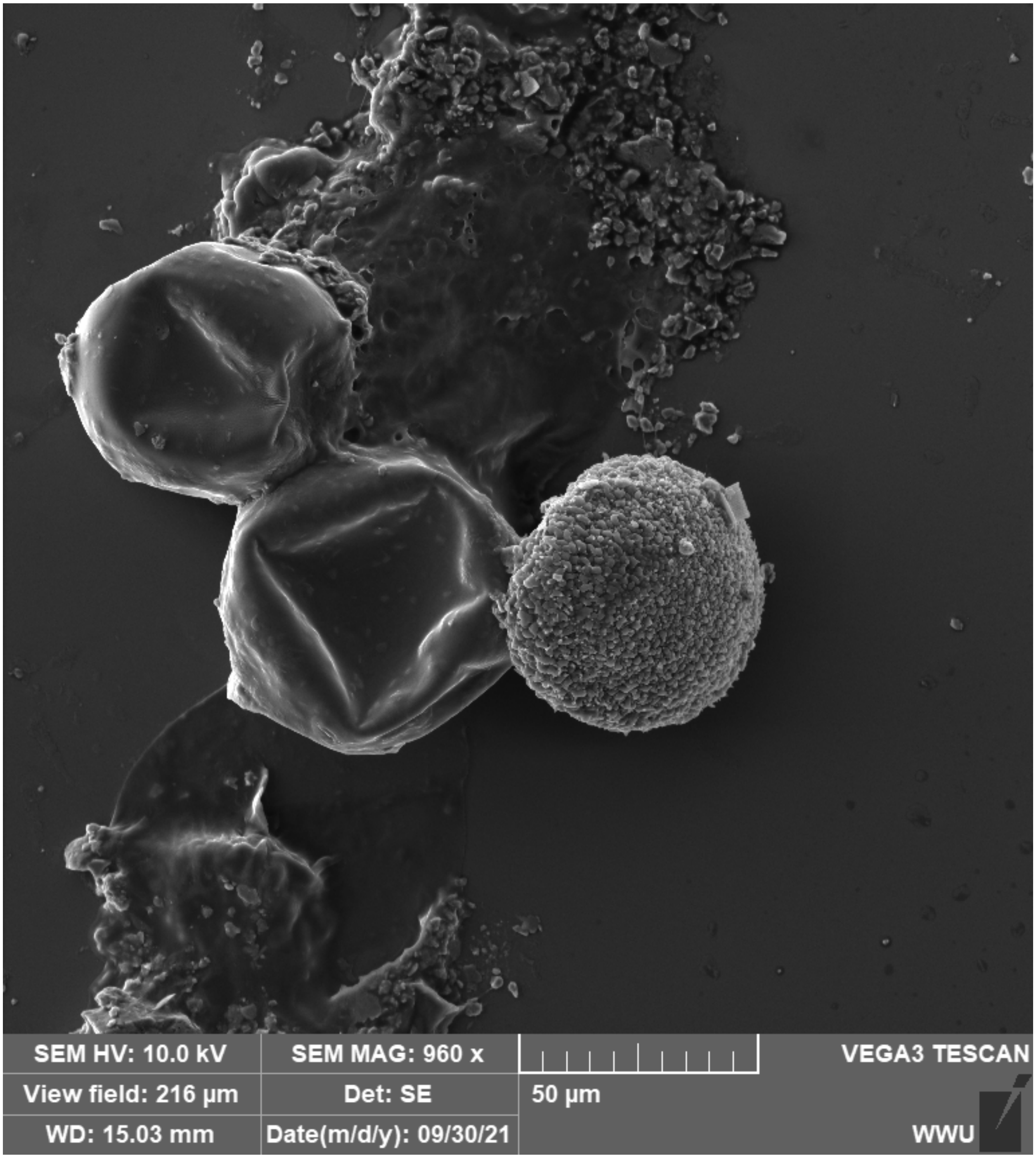

SEM images of cells collected 070621 from site F. Smooth walled cells are vegetative and cell with bumpy, thick cell wall is a cyst. Cells were prepared from meltwater and are surrounded by debris from the natural sample.
