# Supplementary material for: Patchy and Pink: Dynamics of a Chlainomonas sp. (Chlamydomonadales, chlorophyta) algal bloom on Bagley Lake, North Cascades, WA
Source: FEMS Microbiol Ecol. 2023 Sep 7;99(11):fiad106. doi: 10.1093/femsec/fiad106 (PMC10580270; doi:10.1093/femsec/fiad106)
Supplement: fiad106_Supplemental_Files [file fiad106_supplemental_files.zip › Supp_data Supplement_3_Table_of_18S_references.pdf]

Supplement 3: Table of BLAST references for dominant taxa

Hits for major algal taxa and Chyrtids in amplicon data. The hits included in the table were the equivalent top hits, if no 100% match was found. If there was a single 100% match, the single match was included.

***Chlainomonas* 18S hits**	**All 4 ASVs hit the same references**		
**Description**	**Scientific Name**	**Accession**	**% Identity**
Uncultured alga gene for 18S ribosomal RNA, partial sequence, clone: Otu008	uncultured alga	LC371432.1	99.19%
Uncultured alga gene for 18S ribosomal RNA, partial sequence, clone: Otu008	uncultured alga	LC371426.1	99.19%
Chloromonas rubroleosa isolate CC1 small subunit ribosomal RNA gene, partial sequence	Chloromonas rubroleosa	ON324571.1	99.19%
Chlainomonas sp. 190526Oze2R genes for 18S rRNA, ITS1, 5.8S rRNA, ITS2, 28S rRNA, partial and complete sequence	Chlainomonas sp. 190526Oze2R	LC648244.1	99.19%
Uncultured Chloromonas genes for 18S rRNA, ITS1, 5.8S rRNA, ITS2, 28S rRNA, partial and complete sequence, clone: Chloromonas_sp_TA_1	uncultured Chloromonas	AB903004.1	99.19%
Uncultured Chloromonas genes for 18S rRNA, ITS1, 5.8S rRNA, ITS2, 28S rRNA, partial and complete sequence, clone: Chloromonas_sp_TA_3	uncultured Chloromonas	AB902981.1	95.16%
Chlainomonas sp. DL06 18S small subunit ribosomal RNA gene, partial sequence; internal transcribed spacer 1, 5.8S ribosomal RNA gene, and internal transcribed spacer 2, complete sequence; and 26S ribosomal RNA gene, partial sequence	Chlainomonas sp. DL06	MF803743.1	94.35%
Uncultured alga gene for 18S ribosomal RNA, partial sequence, clone: Otu026	uncultured alga	LC371428.1	94.59%
Chlainomonas sp. LP03 18S small subunit ribosomal RNA gene, partial sequence	Chlainomonas sp. LP03	MF803745.1	91.13%
**Chytrid 18S hits**			
**ASV 2 (second dominant sequence variant in dataset)**			
**Description**	**Scientific Name**	**Accession**	**% Identity**
Uncultured Chytridiomycete partial 18S rRNA gene, clone WS 10-E15	uncultured Chytridiomycota	AJ867631.1	100.00%
Uncultured Chytridiomycota clone T5P2AeC07 18S ribosomal RNA gene, partial sequence	uncultured Chytridiomycota	GQ995414.1	96.00%

Supplement 3: Table of BLAST references for dominant taxa

Uncultured Chytridiomycota clone T3P1AeC03 18S ribosomal RNA gene, partial sequence	uncultured Chytridiomycota	GQ995413.1	96.00%
**ASV 11 (second dominant sequence variant in dataset)**			
**Description**	**Scientific Name**	**Accession**	**% Identity**
Uncultured Chytridiomycota clone T5P2AeD09 18S ribosomal RNA gene, partial sequence	uncultured Chytridiomycota	GQ995428.1	98.41%
Uncultured Chytridiomycota clone T2P1AeA09 18S ribosomal RNA gene, partial sequence	uncultured Chytridiomycota	GQ995427.1	98.41%
Uncultured Chytridiomycota clone env_Pavin_epi_T_NS69H 18S ribosomal RNA gene, partial sequence	uncultured Chytridiomycota	JX869380.1	96.83%
Uncultured Chytridiomycota clone env_Pavin_epi_T_NS52C 18S ribosomal RNA gene, partial sequence	uncultured Chytridiomycota	JX869379.1	96.83%
Uncultured Chytridiomycota clone env_Pavin_epi_T_NS610F 18S ribosomal RNA gene, partial sequence	uncultured Chytridiomycota	JX869378.1	96.83%
Uncultured Chytridiomycota clone PA2009B14 18S ribosomal RNA gene, internal transcribed spacer 1, 5.8S ribosomal RNA gene, and internal transcribed spacer 2, complete sequence; and 28S ribosomal RNA gene, partial sequence	uncultured Chytridiomycota	HQ191389.1	96.83%
Uncultured Chytridiomycota clone PA2009B10 18S ribosomal RNA gene, internal transcribed spacer 1, 5.8S ribosomal RNA gene, and internal transcribed spacer 2, complete sequence; and 28S ribosomal RNA gene, partial sequence	uncultured Chytridiomycota	HQ191376.1	96.83%
Uncultured Chytridiomycota clone PA2009C20 18S ribosomal RNA gene, internal transcribed spacer 1, 5.8S ribosomal RNA gene, and internal transcribed spacer 2, complete sequence; and 28S ribosomal RNA gene, partial sequence	uncultured Chytridiomycota	HQ191360.1	96.83%
Uncultured Chytridiomycota clone PA2009A19 18S ribosomal RNA gene, internal transcribed spacer 1, 5.8S ribosomal RNA gene, and internal transcribed spacer 2, complete sequence; and 28S ribosomal RNA gene, partial sequence	uncultured Chytridiomycota	HQ191322.1	96.83%
Uncultured Chytridiomycota clone PA2009A18 18S ribosomal RNA gene, internal transcribed spacer 1, 5.8S ribosomal RNA gene, and internal transcribed spacer 2, complete sequence; and 28S ribosomal RNA gene, partial sequence	uncultured Chytridiomycota	HQ191311.1	96.83%

Supplement 3: Table of BLAST references for dominant taxa

Uncultured Chytridiomycota clone PA2009E23 18S ribosomal RNA gene, internal transcribed spacer 1, 5.8S ribosomal RNA gene, and internal transcribed spacer 2, complete sequence; and 28S ribosomal RNA gene, partial sequence	uncultured Chytridiomycota	HQ191301.1	96.83%
Uncultured Chytridiomycota clone T6P2AeE08 18S ribosomal RNA gene, partial sequence	uncultured Chytridiomycota	GQ995426.1	96.83%
**Uncultured Chyrsophytes 18S hits**			
**ASV 3**			
Uncultured eukaryote isolate ESStr201_76 small subunit ribosomal RNA gene, partial sequence	uncultured eukaryote	MZ300374.1	98.39%
Uncultured eukaryote isolate ESStr278_208 small subunit ribosomal RNA gene, partial sequence	uncultured eukaryote	MZ300653.1	98.39%
Uncultured eukaryote isolate ESStr257_168/300 small subunit ribosomal RNA gene, partial sequence	uncultured eukaryote	MZ297180.1	98.37%
Uncultured Ochromonas clone Esp29 18S ribosomal RNA gene, partial sequence	uncultured Ochromonas	MG674914.1	95.97%
**ASV 8**			
Uncultured Ochromonas clone Esp29 18S ribosomal RNA gene, partial sequence	uncultured Ochromonas	MG674914.1	100%
Uncultured eukaryote clone A4 small subunit ribosomal RNA gene, partial sequence	uncultured eukaryote	AY672809.1	99%
**ASV 16**			
Uncultured Hydrurus sp. clone Boeck31 18S ribosomal RNA gene, partial sequence	uncultured Hydrurus sp.	MG674908.1	100%
Uncultured Hydrurus sp. clone Boeck27 18S ribosomal RNA gene, partial sequence	uncultured Hydrurus sp.	MG674907.1	100%
Uncultured Hydrurus sp. clone Boeck1 18S ribosomal RNA gene, partial sequence	uncultured Hydrurus sp.	MG674904.1	100%
Uncultured eukaryote isolate ESStr292_208 small subunit ribosomal RNA gene, partial sequence	uncultured eukaryote	MZ300668.1	100%
Uncultured eukaryote isolate ESStr266_168 small subunit ribosomal RNA gene, partial sequence	uncultured eukaryote	MZ297189.1	99%
***Chloromonas alpina* 18S hits**			
**ASV 20**			

Supplement 3: Table of BLAST references for dominant taxa

Uncultured Chloromonas genes for 18S rRNA, ITS1, 5.8S rRNA, ITS2, 28S rRNA, partial and complete sequence, clone: Chloromonas_sp_ANT2	uncultured Chloromonas	AB903008.1	100%
Uncultured Chloromonas clone MAL70 18S ribosomal RNA gene, partial sequence	uncultured Chloromonas	JX456234.1	100%
Uncultured Chloromonas clone MAL58 18S ribosomal RNA gene, partial sequence	uncultured Chloromonas	JX456232.1	100%
Chloromonas sp. CCCryo261-06 culture CCCryo<DEU>:261-06 18S ribosomal RNA gene, partial sequence; internal transcribed spacer 1, 5.8S ribosomal RNA gene, and internal transcribed spacer 2, complete sequence; and 26S ribosomal RNA gene, partial sequence	Chloromonas sp. CCCryo261-06	HQ404889.1	100%
Chloromonas sp. CCCryo192-04 culture CCCryo<DEU>:192-04 18S ribosomal RNA gene, partial sequence; internal transcribed spacer 1, 5.8S ribosomal RNA gene, and internal transcribed spacer 2, complete sequence; and 26S ribosomal RNA gene, partial sequence	Chloromonas sp. CCCryo192-04	HQ404880.1	100%
Chloromonas alpina culture CCCryo<DEU>:033-99 18S ribosomal RNA gene, partial sequence; internal transcribed spacer 1, 5.8S ribosomal RNA gene, and internal transcribed spacer 2, complete sequence; and 26S ribosomal RNA gene, partial sequence	Chloromonas alpina	HQ404865.1	100%
Chloromonas alpina strain CCCryo 032-99 18S ribosomal RNA gene, partial sequence; internal transcribed spacer 1, 5.8S ribosomal RNA gene, and internal transcribed spacer 2, complete sequence; and 26S ribosomal RNA gene, partial sequence	Chloromonas alpina	HQ404864.1	100%
**uncultured *Chloromonas* 18S hits**			
**ASV 7**			
Uncultured alga gene for 18S ribosomal RNA, partial sequence, clone: Otu003	uncultured alga	LC371421.1	100.00%
**ASV 9**			
Uncultured alga gene for 18S ribosomal RNA, partial sequence, clone: Otu003	uncultured alga	LC371421.1	100.00%
