# Supplementary figures and images for: Patchy and Pink: Dynamics of a Chlainomonas sp. (Chlamydomonadales, chlorophyta) algal bloom on Bagley Lake, North Cascades, WA
Source: FEMS Microbiol Ecol. 2023 Sep 7;99(11):fiad106. doi: 10.1093/femsec/fiad106 (PMC10580270; doi:10.1093/femsec/fiad106)

**Supplement 1:** Tripod and camera used for color intensity image collection.

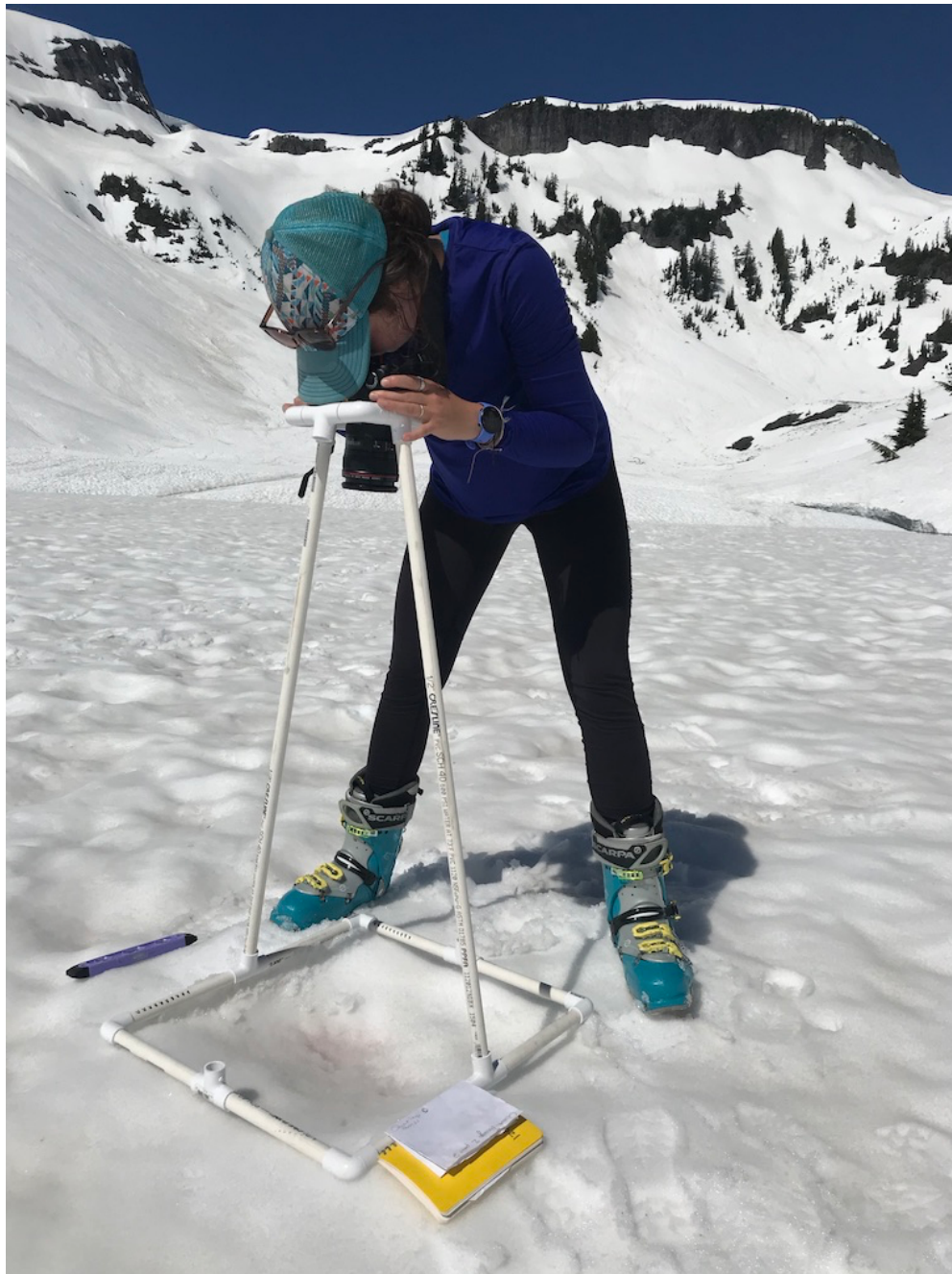

Supplement: fiad106_Supplemental_Files [file fiad106_supplemental_files.zip › Supp_data Supplement_1_Tripod.pdf]
